# Sleep Health Patterns in Romania: Insights from a Nationwide Cross-Sectional Online Survey

**DOI:** 10.3390/brainsci14111086

**Published:** 2024-10-29

**Authors:** Ştefan Strilciuc, Diana Chira, Olivia Verișezan-Roșu, Oana Man-Kesselheim, Oana Stan, Fior Dafin Mureșanu

**Affiliations:** 1 Research Center for Functional Genomics, Biomedicine and Translational Medicine, Iuliu Haţieganu University of Medicine and Pharmacy, 400337 Cluj-Napoca, Romania; 2 RoNeuro Institute for Neurological Research and Diagnostics, 400364 Cluj-Napoca, Romania; olivia.rosu@brainscience.ro (O.V.-R.); oananarcisastan@hotmail.com (O.S.); dafinm@ssnn.ro (F.D.M.); 3 Department of Neuroscience, Iuliu Haţieganu University of Medicine and Pharmacy, 400012 Cluj-Napoca, Romania; 4 Centricity Strategy & Research, 400572 Cluj-Napoca, Romania; oana@centricity.cx

**Keywords:** sleep health, Pittsburgh Sleep Quality Index (PSQI), Romania, online survey

## Abstract

Background: Sleep is one of the most essential processes for sustaining cognitive, emotional, and physical health across all age groups. Insomnia or inadequate sleep significantly impacts health and poses economic burdens due to increased healthcare costs and reduced productivity. Objectives and Methods: This study aimed to investigate sleep quality in the Romanian active population using an online survey incorporating the Pittsburgh Sleep Quality Index (PSQI). Conducted over four months in 2023, the survey gathered 2243 complete responses from urban and rural residents over the age of 18. Results: The results highlight gender and urban–rural disparities in sleep quality, revealing that females and urban residents experienced poorer sleep compared to their counterparts. Additionally, sleep quality was found to significantly worsen with age, with elders (56+ years) reporting the highest PSQI scores, indicating greater sleep difficulties compared to middle-aged adults and youngsters. A high prevalence of sleep disturbances, daytime dysfunctions, and sleep medication use was reported. Common pre-sleep activities included using electronic devices and watching TV, while fewer participants engaged in reading books or consuming alcohol and caffeine. Additionally, participants’ bedding preferences were documented. Conclusions: Our study highlights the influence of various factors on sleep quality and emphasizes the need for targeted public health interventions to improve sleep health in Romania.

## 1. Introduction

Sleep is a vital physiological function for health and well-being in all age groups, from children and adolescents to adults and the elderly. For cognitive, emotional, and mental well-being and the health of the cardiovascular, cerebrovascular, and metabolic systems, adequate sleep is essential, both in terms of quantity and quality [1]. Sleep disorders, like sleep deprivation or insomnia, have a considerable impact on physical and mental health, contributing to behavioral issues in children, cognitive decline, and cardiovascular disease and enhancing the risk of mortality through illnesses and accidents [2,3,4].

Research drawn from demographic studies across various countries indicates that approximately 30% of adults experience one or more symptoms of insomnia. However, these figures can fluctuate based on the specific criteria used to define insomnia and other sleep disturbances [5]. Insomnia is characterized by difficulty falling asleep, staying asleep, or waking up earlier than desired and being unable to return to sleep. These nighttime disturbances are often accompanied by significant daytime impairments, such as fatigue, irritability, and trouble concentrating [6]. In addition to the physical and mental health problems, insomnia also leads to significant economic consequences. These include increased healthcare costs, reduced productivity, and higher rates of absenteeism [7]. Despite their great prevalence and socio-economic strain, sleep problems have received insufficient clinical consideration until recently; they are not only a public health concern, but also an economic priority [8]. 

National epidemiological studies are essential to translate these insights into public health strategies, targeted interventions, and preventative measures. Such studies can provide a clear picture of the prevalence and distribution of sleep disorders, identify the most important risk factors, and highlight disparities in sleep health across different demographic groups. To understand the specific characteristics of sleep in Romania, a national study on the sleep quality of Romanians was initiated. Using a thoroughly developed online questionnaire alongside the Pittsburgh Sleep Quality Index, our research aimed to gather extensive demographic data. We looked at various factors affecting sleep, such as duration and quality, habitual pre-sleep patterns, health-related issues, and lifestyle choices.

The primary objective of this study was to measure the sleep quality in the Romanian active population, also aiming to identify common sleep habits and patterns within the target demographic. Given the lack of national data on this subject, this study aims to serve as a foundational snapshot of sleep quality in Romania.

## 2. Materials and Methods

The study was conducted by the RoNeuro Institute for Neurological Research and Diagnostic between July 24 and 2 October 2023, targeting Romanians over the age of 18 who have access to the internet and who use social media. The study was carried out as an online survey, aiming to leverage the reach and accessibility of digital platforms to engage a broad participant base, acknowledging a tradeoff with the robustness of outcome measurement. The study was designed to provide a descriptive analysis of sleep health patterns and related demographic characteristics in Romania.

The survey was developed by two neurologists specializing in sleep medicine. It consisted of 50 questions, designed to gather extensive demographic data, like age, education level, gender, and urban/rural backgrounds and assess sleep quality using the Pittsburgh Sleep Quality Index (PSQI) and explore factors affecting sleep such as pre-sleep activities, health conditions, and bedding preferences. To ensure clarity and effectiveness, the survey was pre-tested before being conducted via SurveyMonkey.

Participants were required to provide some preliminary data before starting the survey, and individuals under 18 were automatically excluded from proceeding. Those aged 18 and over were presented with the GDPR (General Data Protection Regulation) policy and informed that completing the questionnaire would take approximately 10 min, that it was completely anonymous, and that the data would be used in aggregate; by proceeding, participants provided informed consent, ensuring they were fully aware of the study’s purpose and their involvement. At the end of the survey, participants were asked if they wanted to be involved in future sleep improvement activities and were invited to leave their contact details, preferably an email address, after reviewing the data privacy policy.

The survey was disseminated with the help of Facebook ads, aiming for a representative sample in the active Romanian population, which was approximately 9.069 million people at the time of the survey. The active population likely to be reached was assumed to be 45.4% for rural and 54.6% for urban communities, targeting 5.196 million people in total.

A representative sample size calculated to achieve a 95% confidence level with a 5% margin of error included 385 respondents from both rural and urban areas. For a 3% margin of error, the sample size increased to 1067 respondents from both rural and urban areas, aiming for a total of 770 responses (5% margin) to 2134 responses (3% margin).

The main outcome of the study was the Pittsburgh Sleep Quality Index (PSQI), a validated tool designed to comprehensively measure sleep quality over a 1-month time interval. The PSQI was chosen over other sleep instruments, such as the Epworth Sleepiness Scale (ESS), due to its ability to assess a broader range of sleep-related factors. More exactly, the PSQI questionnaire encompasses various dimensions of sleep health, such as subjective sleep quality, sleep latency, sleep duration, habitual sleep efficiency, sleep disturbances, use of sleeping medication, and daytime dysfunction. It consists of 19 individual items grouped into these seven components, which are then summed to yield a global PSQI score, ranging from 0 to 21, where higher scores indicate poorer sleep quality. A global score of 5 or above is commonly used to distinguish between individuals with “good” or “poor” sleep. This threshold has been established and validated in both clinical and non-clinical populations, making the PSQI a highly suitable tool for assessing sleep patterns in a population-based study [9].

To adapt the PSQI for this study, modifications were made to transition it into an online format, ensuring ease of access and completion for participants. This online adaptation maintained the integrity and validity of the original tool, while enhancing its reach and convenience for a diverse population.

The online survey format improved the study’s reliability and the generalizability of its findings by encouraging broad participation from a variety of demographic backgrounds. Upon collection, the responses were processed to calculate a sleep quality score for each participant. This score, derived from the cumulative assessment of the various dimensions covered by the PSQI, served as the primary variable for evaluating the sleep quality among the study population. The comprehensive nature of the PSQI allowed for a detailed assessment of the respondent’s sleep patterns and overall sleep quality by covering a wide range of sleep-related issues and was an ideal choice for our extensive survey, enabling us to capture a nuanced understanding of sleep health among the Romanian population.

In addition to the PSQI, our study also dived into factors and routines that have the potential to influence sleep quality. We focused on a range of variables such as the types of pillows and bedding used, the materials of these items, and the presence of any health conditions that participants suffer from. We also assessed pre-sleep habits including physical activities/sports, screen time, reading printed books, and consumption habits of coffee and alcohol.

## 3. Results

The survey yielded a total of 2243 full responses and 4276 partial responses, ensuring a representative sample of the target population with a margin of error of 3%. The demographic profile of the study participants can be found in Table 1.

Out of the total respondents, 55.79% identified as female. Conversely, males represented 44.21% of the study’s demographic.

### 3.1. PSQI Scores

Upon analysis of the PSQI scores, differences were noted between genders, as shown in Table 2. Male participants had a mean PSQI score of 4.26 (SD = 4.60), while female participants reported a higher mean score of 5.05 (SD = 4.72). To assess the statistical significance of these differences, an independent t-test was conducted. The results indicate that the difference in PSQI scores between male and female participants was statistically significant (t = 5.41, *p* < 0.0001). Additionally, the Wilcoxon rank-sum test confirmed these findings, showing a statistically significant difference in the distribution of PSQI scores between genders (Z = −5.6700, *p* < 0.0001). Female participants exhibited higher sleep disturbance, as evidenced by their higher PSQI score. These results suggest that women in the study population experience poorer sleep quality compared to men.

Furthermore, an examination of the participants’ backgrounds indicated a notable difference in sleep scores between urban and rural residents, as depicted in Table 3. Participants from rural environments reported a lower mean PSQI score of 3.68 (SD = 4.77), hinting at better sleep quality compared to their urban counterparts, who had a mean score of 4.98 (SD = 4.62). An independent *t*-test was performed, which revealed a significant difference between the PSQI scores of rural and urban participants (t = −7.89, *p* < 0.0001), indicating that urban residents experienced significantly poorer sleep quality compared to rural residents. Additionally, the Wilcoxon rank-sum test further confirmed this significant difference in PSQI scores between urban and rural participants (Z = −9.0044, *p* < 0.0001). The findings suggest that individuals residing in urban areas are more likely to experience sleep disturbances than those in rural settings.

In addition to the disparities observed across gender and urban versus rural backgrounds, our analysis highlights an interesting pattern in sleep quality that varies across different life stages, as illustrated in Table 4. To investigate how sleep quality varies across different stages of life, participants were grouped into three broader age categories: youngsters (18–35 years), middle-aged (36–55 years), and elders (56 years and above). The analysis revealed statistically significant differences in PSQI scores across these three age groups, as shown in Table 4. Youngsters had the lowest mean PSQI score (M = 7.06, SD = 3.32), indicating comparatively better sleep quality. In contrast, elders reported the highest mean PSQI score (M = 8.48, SD = 3.90), reflecting the greatest challenges in maintaining sleep quality. Middle-aged participants fell between the two, with a mean PSQI score of M = 7.76 (SD = 3.64), suggesting moderate difficulties in sleep quality compared to the other groups.

A one-way ANOVA was performed to assess the statistical significance of the observed differences in PSQI scores among these age categories. The results indicate a significant effect of age on sleep quality (F(2, 2468) = 24.07, *p* < 0.0001), confirming that sleep quality deteriorates with age. Post hoc Tukey–Kramer tests confirmed that the differences between each group were statistically significant, with elders experiencing significantly worse sleep quality compared to both the middle-aged and youngster age groups.

The PSQI evaluates sleep quality through several dimensions, each assessing a different aspect of sleep.

- Subjective Sleep Quality—Most participants reported a positive perception of their sleep quality, with a significant majority rating it as either “Very Good” or “Fairly Good”.
- Sleep Latency—The time it takes participants to fall asleep varied, with many reporting prolonged periods. Notably, a considerable number of participants took more than 30 min to fall asleep, highlighting issues with the initiation of sleep.
- Sleep Duration—Regarding sleep duration, less than one-fifth of the sample managed to achieve more than 7 h of sleep per night. The majority slept less than the optimal duration, indicating a prevalent issue with obtaining sufficient sleep among the population.
- Habitual Sleep Efficiency—Habitual sleep efficiency was high in over half of the participants, suggesting that once they fall asleep, they maintain a good sleep. However, the data also reveal that a non-negligible percentage of the population struggles with low or very low sleep efficiency.
- Sleep Disturbances—Sleep disturbances occurred frequently with most participants experiencing them at least once a week.
- Use of Sleep Medication—The reliance on sleep medication was relatively high, with approximately 30% of the participants using medication to aid their sleep at varying frequencies throughout the month. This dependency indicates that a considerable segment of the population experiences difficulties managing sleep naturally.
- Daytime Dysfunction—Daytime dysfunction due to poor sleep was also clearly visible, with the majority of participants reporting some difficulty in daily functioning.

The detailed data presented in Table 5 illustrate the various dimensions of sleep health within the studied population.

### 3.2. Sleep Patterns and Health Insights

In addition to examining sleep quality through the PSQI scores, we aimed to look at the distribution of various sleep-related behaviors, preferences, and health conditions within our sample.

In observing the sleep quality among Romanians, 43.37% of participants reported using electronic devices before bed, while 33.24% watched TV. Taking sleep medication was noted by 17.94%, and 15.25% preferred reading books. Sexual activity was reported by 8.73%, with 30.83% sharing their bed with someone else. Consuming alcohol and coffee or smoking was less common, at 5.78% and 4.96%, respectively, and only 2.53% engaged in working out before sleep. The complete distribution of these sleep behaviors and preferences is presented in Table 6.

Preference for bedding materials shows a clear division in material choices, with a significant majority of participants opting for synthetic materials for their bed linens. When it comes to duvets and pillows, synthetic materials and feathers were preferred. Also, we observed a diversity in pillow types, with feather pillows being the most popular. Mattress preferences varied widely, with memory foam and springs being particularly well liked. The detailed preferences for bedding, including types of materials for linens, duvets, pillows, and mattresses are available in Table 7.

A notable percentage of the sample reported conditions such as allergies and hypertension, which are known to interfere with sleep by causing discomfort and respiratory issues. Additionally, conditions directly related to sleep disturbances, such as sleep apnea, were also present in a portion of our participants. Table 8 provides a breakdown of the self-reported health conditions in this sample.

## 4. Discussion

The aim of this survey was to provide information about sleep quality in the Romanian active population by looking at self-reported characteristics in relation to common sleep habits within the target demographic.

A gender analysis revealed that female participants reported worse sleep quality compared to male participants, as indicated by their higher mean PSQI scores. This suggests that, on average, women in our sample experienced greater difficulties in achieving good quality sleep than men. This trend is not specific for the Romanian population, as other countries, like Austria, Germany, Portugal, and Brazil, report the same disparities between genders [7,10,11,12]. These consistent findings point to possible underlying causes that may have a greater impact on women than on men. In addition to the already demonstrated physical and hormonal changes that impact women’s sleep during their lives [13], other factors, related to work, social, family, and domestic life may contribute to sleep disturbances. For the most part, this is because the recent increase in the number of working-age women has not been followed by a reduction in household responsibilities and family duties [14]. Within this particular socio-cultural setting, women’s sleep has suffered and women may experience prolonged sleep deprivation and negative health outcomes too [15,16]. The psychological burden of balancing all of these roles can exacerbate sleep problems, with studies showing that depressive symptoms can correlate with poor sleep quality in women [16]. This suggests that mental health is an essential factor to consider in understanding sleep disturbances.

Furthermore, when focusing on background, the urban–rural divide could point to the environmental impact on sleep. Urban dwellers could face more challenges in achieving quality sleep than rural dwellers, findings also recorded in other countries like China and Serbia [17,18]. Traffic and ambient noise can elevate stress levels leading to increased sleep disturbances in people living in urban areas [19,20]. Also, air pollution with high levels of carbon dioxide (CO₂) and nighttime light may represent specific urban factors that influence the quality of sleep [21,22]. However, the findings in our study might be strongly influenced by the low percentage of rural residents that responded to our questionnaire (21.64% from rural areas—78.36% from urban areas).

In the Romanian population, sleep quality exhibits significant differences across age groups, as shown by the PSQI scores. Elders (56+ years) report the highest PSQI scores, indicating the greatest difficulties with sleep. Middle-aged adults also face substantial sleep challenges with a mean PSQI score of 7.76, while youngsters (18–35 years) report better sleep quality overall. These findings suggest that sleep quality worsens with age, particularly in the elderly population, aligning with studies from Austria where the PSQI scores increase with age, reaching 8.66 for men over 60 years and 9.80 for women of the same age [7]. In the German population, sleep quality does not deteriorate dramatically with age. Higher scores were reported in the 50–59 age group, with better sleep reported after this age range [10]. Similar findings were also mentioned in the South Korean population [23]. The Portuguese sleep much like the Austrians. There is a slight increase in PSQI scores toward older ages, reaching 6.2 for people in their 70s and 80s [11]. These observations may suggest that middle-aged adults encounter distinct challenges in achieving restful sleep. Potential contributing factors include lifestyle choices, stress levels and anxiety, multiple responsibilities, and health issues. Additionally, for older adults, the decline in sleep quality may be attributed to age-related physiological changes, increased prevalence of chronic health conditions, reduced physical activity, and disruptions in circadian rhythms [24].

Looking at different components of the PSQI scale, detailed insights about the quantity and quality of several aspects of sleep were observed. In Romania, over 97% of the participants rated their sleep quality as “Very Good” or “Fairly Good”. In contrast, only 31% of the Austrian sample described themselves as “good sleepers” [7]. In Taiwan, however, the subjective sleep quality variated across different regions of the country, suggesting the existence of regional factors, such as urbanization and lifestyle, that could play a role in sleep health and quality [7,25]. A significant number of Romanian participants reported needing more than 30 min to fall asleep, with 52.9% of respondents indicating this was the case. These results are consistent with other studies—23% of the Austrian people and 28.9% of the Germans—highlighting prevalent sleep initiation issues across different European populations, possibly influenced by lifestyle or psychological factors [7,10].

Our sleep duration findings resonate with the Austrian data, where less than half of the respondents achieved the recommended 7–9 h of sleep, compared with 18.91% in Romania. Also, the participants with sleep complaints from the Portuguese study often reported a shorter sleep duration. Insufficient sleep duration is a widespread issue across Europe that could contribute to broader public health concerns [7,11]. Habitual sleep efficiency was high in more than 50% of the Romanian participants, with a percentage around 18% of people having “low-efficiency” or “very-low efficiency’’ sleep. Without exact numbers reported, the Portuguese and the Austrian seem to have a lower efficiency due to frequent sleep disturbances [7,11].

The findings concerning sleep disturbances were similar across all studies. The number of sleep disruptions is considerably high in all participants, regardless of their origin, compromising the sleep quality of many individuals [7,10,11,25]. The difficulties in managing sleep are reflected in the use of sleep medication too. In Romania, almost 30% of the people use medication to aid their sleep, as compared to Austria where only 11% of participants reported current use of sleep medication, which is similar to Germany. In Taiwan, the scores are closer to Romania, with 25% of participants using sedatives and hypnotics. Likewise, England reported doubled consumption of hypnotic medication in just seven years [7,10,25,26]. The high prevalence of daytime dysfunctions reported by the participants from all these studies could illustrate the impact of inadequate sleep on daily productivity and quality of life. All these numbers underscore the need for interventions aimed at improving natural sleep and reducing dependency on medication.

In addition to assessing sleep quality, our study sought to find out the pre-sleep habits and preferred activities of Romanians. Not surprisingly, 43.3% of respondents use electronic devices before sleep, while 33.2% of people watch TV. This result is concordant with the Austrian results, where 44.3% of participants use light-emitting electronic devices (laptop, tablet, and phone) right before sleep [7]. Even though research has shown that these activities may disrupt sleep patterns due to the emission of light and sound [27], people prefer these passive and less interactive behaviors. On the other hand, reading a physical book, a well-known method that is associated with improved sleep [28], was chosen only by 15.2% of the participants.

In Romania, few participants reported engaging in behaviors like consuming alcohol, caffeine, or smoking. This may indicate they are aware that these activities may have a negative impact on sleep, especially caffeine consumption and smoking [10,29]. The impact of moderate alcohol consumption on sleep quality, however, remains debatable, with several studies showing that it might be positively associated with a good quality of sleep [10,12,29]. However, these results may be attributed to reporting bias as individuals are less likely to self-report on well-known behaviors that negatively impact health [30].

Another variable of interest was the choice of bedding by materials, as this may also influence sleep [31,32]. Our results show that Romanians prefer synthetic materials for bed linens, suggesting that practical benefits like durability and value for money are prioritized over the natural breathability and temperature regulation that natural materials can offer. Synthetic materials are the most preferred ones when it comes to duvets as well, with a small percentage of people that use wool for their duvets or pillows. However, the importance of comfort and the desire to have optimal support for their bodies is reflected in the use of orthopedic pillows and memory foam mattresses, similar to the Australians, who also consider comfort the most important aspect when choosing a mattress or other types of bedding [33].

Regarding the presence of various health conditions in our participants, the most frequent problems reported were allergies, which are known for interfering with sleep due to discomfort and respiratory issues [34] and hypertension, which can reduce sleep quality due to increased nighttime arousal [35]. Sleep apnea was also described by some of the participants, known for being detrimental due to its direct interference with breathing patterns during sleep [36].

Although our study offers first insights at a national level regarding the quality of sleep in Romania, this study has some limitations which must be acknowledged. Sleep quality is inherently subjective, so participants might not accurately remember their sleep patterns and might be influenced by the timing of the survey, capturing sleep quality at a single point in time. Moreover, people might interpret survey questions differently and they might also provide answers that are more socially acceptable, rather than their true experiences and habits. Also, the validity of the PSQI scale in an online setting might be arguable. Another limitation is related to the mode of data collection. The survey was conducted online and disseminated via social media, primarily Facebook, which could introduce biases in the representativeness of the sample. Populations with limited internet access, such as those in rural areas or older adults who are not frequent social media users, may be under-represented. Consequently, this limitation may affect the generalizability of the findings to these demographic groups.

## 5. Conclusions

Despite these limitations, this study serves as a valuable starting point for understanding sleep health in Romania, providing important demographic data and perspectives and highlighting several potential factors that could affect sleep quality. This study also underscores the need for more comprehensive research to fully understand the complexities of sleep health and quality, as well as the dynamics between the PSQI scores and Romanian’s pre-sleep habits Addressing these issues is important not only for improving individual health and well-being, but also for mitigating the wider societal effects of sleep disturbances. Furthermore, this work serves as a foundational step toward establishing national benchmarks for sleep health in Romania, as it currently is a subject that has not been sufficiently explored, despite its importance. Future studies should delve deeper into the relationships between variables in order to explore how lifestyle choices and health conditions interact to influence sleep patterns.

## Figures and Tables

**Table 1:** Sociodemographic data from study participants.

		Total	Female		Male	
		*n*	*n*	*%*	*n*	*%*
Age	18–25	403	205	50.8	198	49.1
	26–35	661	372	56.2	289	43.7
	36–45	798	456	57.1	342	42.8
	46–55	949	563	59.3	386	40.6
	56–65	1054	567	53.7	487	46.2
	66–75	242	146	60.3	96	39.6
	>75	56	25	44.6	31	55.3
Marital Status	Married	1108	693	62.5	415	37.4
	Divorced	141	93	65.9	48	34.0
	In a relationship	501	303	60.4	198	39.5
	Single	374	172	45.9	202	54.0
	Widowed	65	55	84.6	10	15.3
	Unknown	38	22	57.8	16	42.1
Background	Urban	3350	1947	54.8	1403	41.8
	Rural	952	438	46.0	487	51.1
Level of Education	No diploma	8	2	25.0	6	75.0
	Primary education	219	94	42.9	125	57.0
	High school	1409	698	49.5	711	50.4
	University degree	1584	925	58.3	659	41.6
	Postgraduate	1018	625	61.3	366	35.9

**Table 2:** PSQI comparisons by gender.

Gender	Mean	SD	Mean Difference (95% CI)	t-Value	*p*-Value	Wilcoxon Z	*p*-Value (Wilcoxon)
Female	5.05	4.72	0.7824 (0.5001, 1.0648)	5.41	<0.0001	−5.6700	<0.0001
Male	4.26	4.60					

**Table 3:** PSQI comparisons by background.

Background	Mean	SD	Mean Difference (95% CI)	t-Value	*p*-Value	Wilcoxon Z	*p*-Value (Wilcoxon)
Urban	3.68	4.77	−1.3687 (−1.7088, −1.0285)	−7.89	<0.0001	−9.0044	<0.0001
Rural	4.98	4.62					

**Table 4:** PSQI scores by age group.

Age Group	Mean	SD	*p*-Value (Tukey–Kramer)
Youngsters	7.06	3.32	*p* = 0.0005
Middle-aged	7.76	3.64	
Elders	8.48	3.90	vs. Youngsters—*p* < 0.0001 vs. Middle-aged—*p* = 0.0005

**Table 5:** Distribution of scores across PSQI subcomponents.

PSQI Components		*n*	*%*
Subjective sleep quality	Very good	1406	54.60
	Fairly good	1109	43.07
	Fairly bad	60	2.33
Sleep latency	Less than 15 min	397	15.42
	16–30 min	814	31.61
	31–60 min	881	34.21
	More than 60 min	438	18.76
Sleep duration	More than 7 h	486	18.91
	6–7 h	762	29.65
	5–6 h	676	26.30
	Less than 5 h	646	25.14
Habitual sleep efficiency	High-efficiency sleep	1494	58.87
	Moderate-efficiency sleep	544	21.43
	Low-efficiency sleep	253	9.97
	Very-low-efficiency sleep	247	9.73
Sleep disturbances	Less than once a week	19	0.74
	Once or twice a week	1387	53.86
	Three or four times a week	1109	43.07
	Five or more times a week	60	2.33
Use of sleep medication	Not during the past month	1798	70.10
	Less than once a week	180	7.02
	Once or twice a week	160	6.24
	Three or more times a week	427	16.65
Daytime dysfunction	No problems at all	353	13.98
	A few problems	1426	56.48
	Some problems	582	23.05
	Big problems	164	6.50

**Table 6:** Distribution of self-reported behaviors before sleep.

Behaviors	*n*	*%*
Reading book	652	15.25
Watching TV	1421	33.24
Using other electronic devices	1854	43.37
Consuming alcohol	242	5.78
Consuming coffee or smoking	212	4.96
Working out	108	2.53
Taking sleep medication	767	17.94
Sharing bed with a partner	1318	30.83
Engaging in sexual activity	373	8.73

**Table 7:** Distribution of preferences for bedding materials.

Bedding Preferences		*n*	*%*
Material	Natural	1542	36.06
	Synthetic	2734	63.94
Duvet type	Wool	263	19.07
	Feathers	418	30.31
	Synthetic	698	50.62
Pillow type	Wool	143	11.36
	Feathers	625	49.64
	Orthopedic	491	39.00
Mattress type	Foam	272	11.03
	Memory Foam	627	25.43
	Springs	653	26.48
	Orthopedic	329	13.34
	Latex	79	3.20

**Table 8:** Distribution of self-reported health conditions in the study sample.

Health Conditions	*n*	*%*
Allergies/Intolerances	534	12.48
Hypertension	456	10.66
Memory problems	185	4.33
Sleep apnea	160	5.26
Diabetes	126	4.19
Heart disease	123	4.09

## Data Availability

Data are available upon reasonable request. The data are not publicly available due to privacy reasons.

## References

[1] Hirshkowitz M., Whiton K., Albert S.M., Alessi C., Bruni O., DonCarlos L., Hazen N., Herman J., Katz E.S., Kheirandish-Gozal L. (2015). National Sleep Foundation’s Sleep Time Duration Recommendations: Methodology and Results Summary. Sleep. Health.

[2] Ramar K., Malhotra R.K., Carden K.A., Martin J.L., Abbasi-Feinberg F., Aurora R.N., Kapur V.K., Olson E.J., Rosen C.L., Rowley J.A. (2021). Sleep Is Essential to Health: An American Academy of Sleep Medicine Position Statement. J. Clin. Sleep. Med..

[3] Perlis M.L., Posner D., Riemann D., Bastien C.H., Teel J., Thase M. (2022). Insomnia. Lancet.

[4] Lim D.C., Najafi A., Afifi L., Bassetti C.L., Buysse D.J., Han F., Högl B., Melaku Y.A., Morin C.M., Pack A.I. (2023). The Need to Promote Sleep Health in Public Health Agendas across the Globe. Lancet Public. Health.

[5] Roth T. (2007). Insomnia: Definition, Prevalence, Etiology, and Consequences. J. Clin. Sleep. Med..

[6] Riemann D., Espie C.A., Altena E., Arnardottir E.S., Baglioni C., Bassetti C.L.A., Bastien C., Berzina N., Bjorvatn B., Dikeos D. (2023). The European Insomnia Guideline: An Update on the Diagnosis and Treatment of Insomnia 2023. J. Sleep Res..

[7] Blume C., Hauser T., Gruber W.R., Heib D.P., Winkler T., Schabus M. (2020). “How Does Austria Sleep?” Self-Reported Sleep Habits and Complaints in an Online Survey. Sleep. Breath..

[8] Sateia M.J., Doghramji K., Hauri P.J., Morin C.M. (2000). Evaluation of Chronic Insomnia. An American Academy of Sleep Medicine Review. Sleep.

[9] Buysse D.J., Reynolds C.F., Monk T.H., Berman S.R., Kupfer D.J. (1989). The Pittsburgh Sleep Quality Index: A New Instrument for Psychiatric Practice and Research. Psychiatry Res..

[10] Hinz A., Glaesmer H., Brähler E., Löffler M., Engel C., Enzenbach C., Hegerl U., Sander C. (2017). Sleep Quality in the General Population: Psychometric Properties of the Pittsburgh Sleep Quality Index, Derived from a German Community Sample of 9284 People. Sleep Med.

[11] Gomes A.A., Marques D.R., Meiavia A.M., Cunha F., Clemente V. (2018). Psychometric Properties and Accuracy of the European Portuguese Version of the Pittsburgh Sleep Quality Index in Clinical and Non-Clinical Samples. Sleep Biol. Rhythm..

[12] Canever J.B., Cândido L.M., Moreira B.d.S., Danielewicz A.L., Cimarosti H.I., Lima-Costa M.F., Avelar N.C.P. (2023). de A Nationwide Study on Sleep Complaints and Associated Factors in Older Adults: ELSI-Brazil. Cad. Saude Publica.

[13] Dorsey A., de Lecea L., Jennings K.J. (2021). Neurobiological and Hormonal Mechanisms Regulating Women’s Sleep. Front. Neurosci..

[14] Lee J., Oh J., Park H., Sim J., Lee J., Kim Y., Yun B. (2023). Exploring the Relationship between Work–Family Conflict and Sleep Disturbance: A Study on Stratification and Interaction. Front. Psychol..

[15] Frange C., Banzoli C.V., Colombo A.E., Siegler M., Coelho G., Bezerra A.G., Csermak M., Naufel M.F., Cesar-Netto C., Andersen M.L. (2017). Women’s Sleep Disorders: Integrative Care. Sleep. Sci..

[16] Mohamed B.E.S., Ghaith R.F.A.H., Ahmed H.A.A. (2022). Relationship between Work–Family Conflict, Sleep Quality, and Depressive Symptoms among Mental Health Nurses. Middle East. Curr. Psychiatry.

[17] Zheng W., Luo X.-N., Li H.-Y., Ke X.-Y., Dai Q., Zhang C.-J., Zhang X.-Y., Ning Y.-P. (2018). Regional Differences in the Risk of Insomnia Symptoms among Patients from General Hospital Outpatient Clinics. Neuropsychiatr. Dis. Treat..

[18] Jakovljević B., Belojević G., Paunović K., Stojanov V. (2006). Road Traffic Noise and Sleep Disturbances in an Urban Population: Cross-Sectional Study. Croat. Med. J..

[19] Halonen J.I., Vahtera J., Stansfeld S., Yli-Tuomi T., Salo P., Pentti J., Kivimäki M., Lanki T. (2012). Associations between Nighttime Traffic Noise and Sleep: The Finnish Public Sector Study. Environ. Health Perspect..

[20] Theakston F., WHO, Weltgesundheitsorganisation (2011). Burden of Disease from Environmental Noise: Quantification of Healthy Life Years Lost in Europe.

[21] Zanobetti A., Redline S., Schwartz J., Rosen D., Patel S., O’Connor G.T., Lebowitz M., Coull B.A., Gold D.R. (2010). Associations of PM10 with Sleep and Sleep-Disordered Breathing in Adults from Seven U.S. Urban Areas. Am. J. Respir. Crit. Care Med..

[22] Chepesiuk R. (2009). Missing the Dark: Health Effects of Light Pollution. Environ. Health Perspect..

[23] Ahn E., Baek Y., Park J.-E., Lee S., Jin H.-J. (2024). Elevated Prevalence and Treatment of Sleep Disorders from 2011 to 2020: A Nationwide Population-Based Retrospective Cohort Study in Korea. BMJ Open.

[24] Tatineny P., Shafi F., Gohar A., Bhat A. (2020). Sleep in the Elderly. Mo. Med..

[25] Tai S.-Y., Wang W.-F., Yang Y.-H. (2015). Current Status of Sleep Quality in Taiwan: A Nationwide Walk-in Survey. Ann. Gen. Psychiatry.

[26] Calem M., Bisla J., Begum A., Dewey M., Bebbington P.E., Brugha T., Cooper C., Jenkins R., Lindesay J., McManus S. (2012). Increased Prevalence of Insomnia and Changes in Hypnotics Use in England over 15 Years: Analysis of the 1993, 2000, and 2007 National Psychiatric Morbidity Surveys. Sleep.

[27] Chang A.-M., Aeschbach D., Duffy J.F., Czeisler C.A. (2015). Evening Use of Light-Emitting eReaders Negatively Affects Sleep, Circadian Timing, and next-Morning Alertness. Proc. Natl. Acad. Sci. USA.

[28] Finucane E., O’Brien A., Treweek S., Newell J., Das K., Chapman S., Wicks P., Galvin S., Healy P., Biesty L. (2021). Does Reading a Book in Bed Make a Difference to Sleep in Comparison to Not Reading a Book in Bed? The People’s Trial—An Online, Pragmatic, Randomised Trial. Trials.

[29] Leger D., Andler R., Richard J.-B., Nguyen-Thanh V., Collin O., Chennaoui M., Metlaine A. (2022). Sleep, Substance Misuse and Addictions: A Nationwide Observational Survey on Smoking, Alcohol, Cannabis and Sleep in 12,637 Adults. J. Sleep. Res..

[30] Hurley L.L., Taylor R.E., Tizabi Y. (2012). Positive and Negative Effects of Alcohol and Nicotine and Their Interactions: A Mechanistic Review. Neurotox. Res..

[31] Chow C.M., Shin M., Mahar T.J., Halaki M., Ireland A. (2019). The Impact of Sleepwear Fiber Type on Sleep Quality under Warm Ambient Conditions. Nat. Sci. Sleep.

[32] Bolton R., Hulshof H., Daanen H.A.M., van Dieën J.H. (2022). Effects of Mattress Support on Sleeping Position and Low-Back Pain. Sleep Sci. Pract..

[33] Bedbuyer Australia’s Largest Sleep and Bedding Survey (in 2020)|Bedbuyer^TM^. https://bedbuyer.com.au/sleep-and-bedding-survey/.

[34] Liu J., Zhang X., Zhao Y., Wang Y. (2020). The Association between Allergic Rhinitis and Sleep: A Systematic Review and Meta-Analysis of Observational Studies. PLoS ONE.

[35] Bock J.M., Vungarala S., Covassin N., Somers V.K. (2022). Sleep Duration and Hypertension: Epidemiological Evidence and Underlying Mechanisms. Am. J. Hypertens..

[36] McKeown P., O’Connor-Reina C., Plaza G. (2021). Breathing Re-Education and Phenotypes of Sleep Apnea: A Review. J. Clin. Med..

